# Silver and Copper Nanoparticles Inhibit Biofilm Formation by Mastitis Pathogens

**DOI:** 10.3390/ani11071884

**Published:** 2021-06-24

**Authors:** Agata Lange, Agnieszka Grzenia, Mateusz Wierzbicki, Barbara Strojny-Cieslak, Aleksandra Kalińska, Marcin Gołębiewski, Daniel Radzikowski, Ewa Sawosz, Sławomir Jaworski

**Affiliations:** 1 Department of Nanobiotechnology, Institute of Biology, Warsaw University of Life Sciences, 02-786 Warsaw, Poland; agata_lange@sggw.edu.pl (A.L.); agnieszka.grzenia22@gmail.com (A.G.); mateusz_wierzbicki@sggw.edu.pl (M.W.); barbara_strojny@sggw.edu.pl (B.S.-C.); ewa_sawosz_chwalibog@sggw.edu.pl (E.S.); 2 Department of Animal Breeding, Institute of Animal Sciences, Warsaw University of Life Sciences, 02-786 Warsaw, Poland; aleksandra_kalinska1@sggw.edu.pl (A.K.); marcin_golebiewski@sggw.edu.pl (M.G.); daniel_radzikowski@sggw.edu.pl (D.R.)

**Keywords:** biofilm, mastitis pathogens, bovine mastitis, silver nanoparticles, copper nanoparticles, silver-copper complex

## Abstract

**Simple Summary:**

Bovine mastitis is a common disease in cows. It is caused by many pathogen species, which can form three-dimensional structures composed of bacterial cells, known as biofilms. These structures are almost impermeable to antimicrobials, making treatment difficult. We looked at the influence of metal nanometre-scale particles on biofilm formation by several pathogen species. We analysed the properties of these nanoparticles, determined the concentration needed to inhibit the growth of pathogens and to damage their membranes, and finally, checked how nanoparticles influence biofilm formation. We show that metal nanoparticles (silver and copper nanoparticles and their mixture) limit the formation of biofilm very effectively. These results mean that nanoparticles can be used to cure cattle suffering from mastitis, which will lead to higher milk production and less financial loss.

**Abstract:**

Bovine mastitis is a common bovine disease, frequently affecting whole herds of cattle. It is often caused by resistant microbes that can create a biofilm structure. The rapidly developing scientific discipline known as nanobiotechnology may help treat this illness, thanks to the extraordinary properties of nanoparticles. The aim of the study was to investigate the inhibition of biofilms created by mastitis pathogens after treatment with silver and copper nanoparticles, both individually and in combination. We defined the physicochemical properties and minimal inhibitory concentration of the nanoparticles and observed their interaction with the cell membrane, as well as the extent of biofilm reduction. The results show that the silver–copper complex was the most active of all nanomaterials tested (biofilm was reduced by nearly 100% at a concentration of 200 ppm for each microorganism species tested). However, silver nanoparticles were also effective individually (biofilm was also reduced by nearly 100% at a concentration of 200 ppm, but at concentrations of 50 and 100 ppm, the extent of reduction was lower than for the complex). Nanoparticles can be used in new alternative therapies to treat bovine mastitis.

## 1. Introduction

### 1.1. Biofilms

A biofilm is a multicellular structure with specific composition, formed by microorganisms. In addition to bacterial cells, biofilm contains water and extracellular polymeric substance (EPS), which consists mainly of polysaccharides, proteins, nucleic acids, and surfactants [1]. It allows microorganisms to adhere particularly strongly to both biotic and abiotic surfaces, especially when exposed to unfavourable environmental factors, and hence biofilm forming is recognised as protection against damage [2]. The exopolysaccharide matrix may also be impermeable to antimicrobials and inhibit their penetration into the biofilm [3].

Maturation of the biofilm includes steps such as initial reversible and irreversible attachments, maturation, and dispersion. The last step is critical, allowing the biofilm to embed in new areas through detachment of planktonic forms, which initiate new biofilm structures in other locations [4].

The phenotype structure of a biofilm, regardless of its shape, is always similar. Small cells with limited multiplication potential are located inside the bacterial community, whereas outward-facing areas are occupied by metabolically active cells. This is due to the reduced availability of oxygen and nutrients in the centre of the structure [5]. Bacterial heterogeneity may promote the development of various resistant features, which, as a result, may encompass the whole community [3]. Remarkably, bacteria without resistant features initially become less sensitive to antimicrobials when grown in a biofilm structure [6]. “Quorum sensing” is the main means of communication in bacterial clusters, where every cell produces chemical compounds (such as bacteriocins), and certain genes are expressed. This phenomenon is considered a resistant mechanism [7]. The larger the population of microorganisms, the more self-inducing compounds are secreted. Changes in gene expression occur, and eventually the whole population is altered [8]. The prevalence of resistant bacteria in biofilms is one of the reasons bacterial infections are difficult to treat. The mechanisms of medications may also be ineffective against bacteria embedded in a biofilm [9]. Initially, infections are usually easily treatable because of the sensitivity of the planktonic forms that cause them. The difficulty arises when infections become chronic. They become much tougher to combat because by then they form advanced biofilm structures [10].

### 1.2. Bovine Mastitis

Bovine mastitis, caused by many bacterial species, is one of the most common bovine illnesses, affecting whole herds of cattle [10]. This affects the amount of milk produced, which in turn leads to financial losses in the dairy industry due to low productivity [11].

Mastitis pathogens are characterised as either contagious (spreading through the milking process) or environmental microorganisms. The contagious group includes *Staphylococcus aureus* and *Streptococcus agalactiae*, whereas the main environmental pathogens are *Escherichia coli*, *Streptococcus dysgalactiae*, and other streptococci [12]. While more than 130 bacterial species can cause the illness, *S. aureus* is one of the most common causes of chronic mastitis, because it can form a biofilm structure [13]. The biofilm-forming process allows bacteria such as staphylococci to colonise the internal part of the udder, and in particular, the biofilm structure with a polymeric matrix allows microbials to survive antimicrobial treatments [14]. Likewise, *E. faecalis*, which also creates a biofilm structure, has additional inherent resistance to certain antimicrobials, making it even more challenging to eradicate [15].

The rise in the number of resistant bacteria in the dairy industry is related to frequent use of antibiotics on herds of cattle. These pathogens are not thought to be dangerous to humans if only pasteurised milk is consumed, but they may still constitute a health hazard to the human population, especially since an increasing number of people consume raw milk [16]. Furthermore, the detrimental influence of mastitis on cattle health and milk production emphasises the urgent need for effective strategies to prevent and control the development of the disease [11].

### 1.3. Nanoparticles

Nanoparticles are widely used in biology and medicine due to their ability to freely penetrate the organisms’ barriers [17]. The most popular medical nanomaterial is silver nanoparticles (AgNPs), which have been used as a component of antiseptics since ancient times because of their remarkable antibacterial properties and relatively low toxicity [18]. Nanosilver is thought to be effective against many of the resistant pathogens, and may become an alternative to antibiotics in the future. Nanoparticles interact with bacterial cells in several ways, including by disturbing cell layers and generating reactive oxygen species that damage internal structures [19]. Silver ions interact with the outer membrane (or wall), depriving bacteria of protection against harmful external factors [20]. They are also able to intercalate into nucleic acids, as well as to disturb ribosomes, which may lead to the inhibition of basic life processes in a cell, such as transcription and translation [20]. Nanoparticles with a diameter greater than 10 nm interact with the cell wall and membrane, causing its disintegration and cell death [21].

Copper nanoparticles (CuNPs), characterised by a high surface to volume ratio, also have great potential as antimicrobials due to easy surface functionalisation with other compounds to amplify the primary antibacterial effect [22]. According to a previous study [23], a combination of copper nanoparticles with carbon nanotubes inhibits the growth of the bacterial biofilm of *Methylobacterium* spp. and is not toxic to human fibroblasts. Similar effects have been observed for highly resistant *Pseudomonas aeruginosa* treated with copper nanoparticles [24]. Several nanomaterials exhibit tremendous antibacterial properties and inhibit biofilm formation on various surfaces for an extended period [9]. However, although nanoparticles are known to damage cells and to penetrate and disrupt biofilms, their precise mechanism of action is not fully understood [2].

### 1.4. Objective

The aim of the study was to investigate the ability of silver and copper nanoparticles both together and separately to inhibit biofilm formation produced by mastitis pathogens.

## 2. Materials and Methods

### 2.1. Nanoparticles

AgNPs and CuNPs were obtained from aXonnite (Nano-Tech, Warsaw, Poland). Silver-copper (Ag-Cu) complexes were prepared by mixing 50 ppm of the hydrocolloids of each of the nanoparticles in a 1:1 ratio, and the obtained mixture was sonicated for 45 min at room temperature. Prior to use in experiments, each compound was subjected to ultrasonic treatment for 30 min.

### 2.2. Physicochemical Analysis

Physicochemical analysis was conducted at room temperature (25 °C). The dynamic light scattering method was used for size distribution, and laser Doppler electrophoresis was used for zeta potential analysis with the Zetasizer Nano-ZS ZEN 3600 (Malvern, Malvern Town, UK). For morphology analysis, transmission electron microscopy with a voltage of 80 keV was used. 10 μL of nanoparticles was applied to copper grids (Mesh Cu Grids, Agar Scientific, Stansted, UK) and dried. Samples were observed under a microscope (JEM-1220, JEOL, Tokyo, Japan).

### 2.3. Bacterial Strains

Bacterial strains *Streptococcus agalactiae* (ATCC-31475), *Streptococcus dysgalactiae* (ATCC-12388), *Enterococcus faecalis* (ATCC-47077), *Staphylococcus aureus* (ATCC-27821), *Salmonella Enteritidis* (ATCC-BAA-1734), *Escherichia coli* (ATCC-12814), and *Enterobacter cloacae* (ATCC-35030), as well as yeast *Candida albicans* (ATCC-24433), were purchased from LGC Standards (Lomianki, Poland). The Mueller-Hinton (MH) broth used for growth and maintenance of the bacterial cultures was supplied by Biomaxima (cat. PS15, Lublin, Poland), whereas the yeast nitrogen base (YNB) for yeast was supplied by Merck Millipore (cat. 51483, Darmstadt, Germany).

### 2.4. Microbial Cultures

Each microbial strain was stored as a suspension in 20% (*v*/*v*) glycerol at −20 °C. Prior to experiments, the glycerol was removed and the microbial cells were washed with distilled water. Then microbial cultures were grown in media with an optimal availability of nutrients: MH broth for bacteria and YNB for yeast. Then the microbial cultures were kept in a bacterial incubator (NUAire, Plymouth, MN, USA) under standard conditions (37 °C).

### 2.5. The Minimal Inhibitory Concentration (MIC) Test

The first step of the MIC test was the preparation of microbial cell dilutions with an optical density (OD) of 0.1, which is equivalent to 10^6^ cells per millilitre. Optical density was measured at a wavelength of 660 nm. For this purpose, 100 μL of the overnight microbial culture was added to 20 mL of liquid medium (MH for bacteria and YNB for yeast). The resulting suspension was then diluted again, yielding the final concentration of 2 × 10^4^ cells per mL. Serial dilution was carried out in a 96-well plate in the presence of a blank control (medium without cells or nanoparticles) and a growth control group (inoculum without nanoparticles). After 24 h of incubation at 30 °C, a reading was taken using a microplate reader (Tecan M200 Infinite, Monachium, Germany; absorbance at 600 nm). This allowed us to select the appropriate concentrations to continue the research. The concentrations selected were 50 ppm, 100 ppm, and 200 ppm, for silver, copper, and silver-copper complex, respectively.

### 2.6. Membrane Integrity

To evaluate cell membrane integrity, the lactate dehydrogenase (LDH) activity was examined using a Cytotoxicity Detection Kit (In Vitro Toxicology Assay Kit, based on lactic dehydrogenase, LDH, Sigma-Aldrich, Hamburg, Germany). 100 μL of bacteria and yeast cells (1 × 10^6^ CFU/mL) were cultured in liquid medium (MH for bacteria and YNB for yeast) on 96-well plates, with the addition of nanoparticles in the concentrations identified as the minimal inhibitory concentration for microbial species used (3.125, 6.25, 12.5, 25 ppm). After 24 h of incubation, 100 μL of the LDH assay mixture was added to each well. The plates were kept in the dark and incubated for 30 min at room temperature (25 °C). The absorbance was recorded at 490 nm on an ELISA reader (Infinite M200, Tecan, Männedorf, Switzerland). LDH leakage was expressed as a percentage of the test sample (reduced by the value of the blank) in relation to the control sample (also reduced by the value of the blank), where a blank probe was the medium without cells, and the control sample was inoculum treated with 100 μL of Triton X-100 (Sigma-Aldrich, Hamburg, Germany).

### 2.7. Biofilm Formation

Hydrocolloids of the nanoparticles at the selected concentrations (50, 100, and 200 ppm) were affixed to wells in a 96-well plate, and the prepared plate was left under the laminar flow cabinet for 24 h until completely dry. After this, 100 µL of microbial culture (1.5 × 10^8^ CFU/mL) was added to each well, and the plate was incubated again for 24 h at 37 °C in a microbiological incubator (NUAire, Plymouth, MN, USA).

Planktonic cells were removed carefully by pipetting the liquid culture from the plate, leaving only the attached biofilm, which was fixed for 5 min with a 2.5% glutaraldehyde solution to inhibit further growth. The fixative was then removed and the wells were washed three times with sterile phosphate-buffered saline (PBS; Sigma-Aldrich, Darmstadt, Germany). To determine the exact quantity of biofilm, cells were stained with 100 μL of 0.25% crystal violet dye and washed gently three times with sterile PBS to remove any additional unbound dye. Plates were dried overnight, and crystal violet was then extracted using a 1:1 acetone:ethanol solution. The biofilm formation level was determined by measuring absorbance at a wavelength of 570 nm (microplate reader Tecan M200 Infinite, Monachium, Germany), which was related to the amount of dye attached to cells compared to controls (cultured in uncoated wells).

### 2.8. Data Analysis

The results were analysed by one-way analysis of variance (ANOVA) with Statgraphics Plus 4.1 (StatPoint Technologies Inc., Warrenton, VA, USA). All data were compiled with ANOVA (conforming to the assumptions), and differences were assumed to be statistically significant at *p*  ≤  0.05.

## 3. Results

### 3.1. Physicochemical Analysis

**Figure 1:**
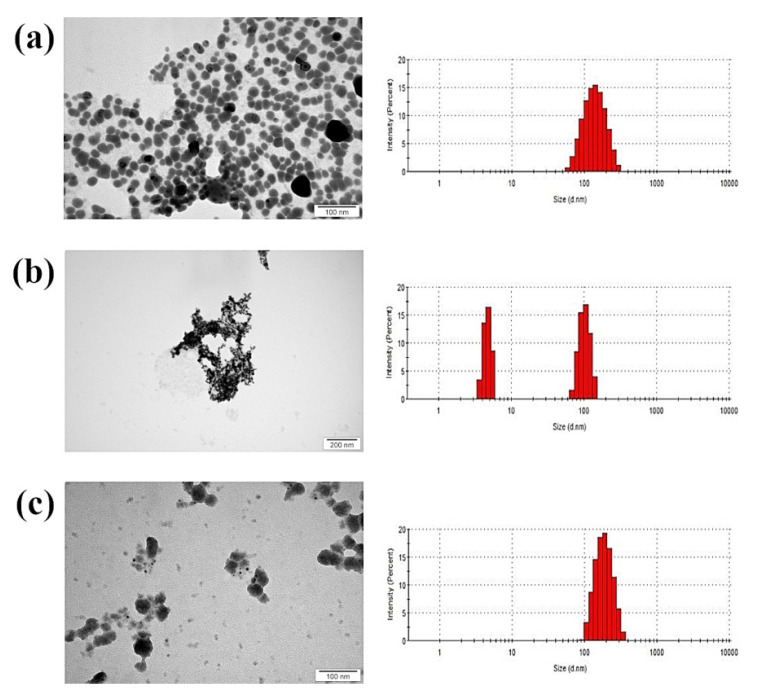
TEM images and size distribution (hydrodynamic diameter) of nanoparticles: (**a**) silver nanoparticles; (**b**) copper nanoparticles; (**c**) silver–copper complex.

AgNPs had the smallest average size and a spherical structure (Figure 1). The Ag-Cu complex showed a mean value of hydrodynamic diameter among tested samples. CuNPs had the biggest average diameter of over 300 nm, although size distribution suggests that two fractions were present, one with nanoparticles smaller than 100 nm, and the other much bigger. However, transmission electron microscopy (TEM) showed that small particles had agglomerated into large structures according to size distribution.

Zeta potential values also confirm the tendency of CuNPs to agglomerate, as the value was close to zero (Table 1). Nevertheless, all samples analysed had a negative zeta potential not exceeding ±30 mV and no colloidal stability.

### 3.2. Minimal Inhibitory Concentration

**Figure 2:**
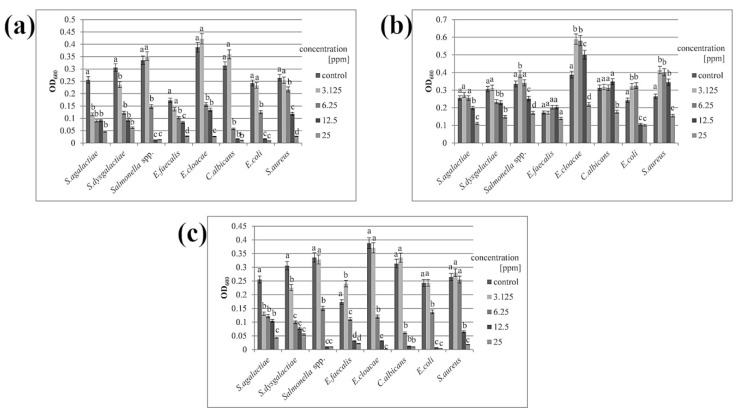
Optical density of microbial growth. Microbes were treated with (**a**) silver nanoparticles; (**b**) copper nanoparticles; (**c**) silver–copper complex. All samples were measured in triplicate and the results were averaged. Columns labelled a–d indicate statistically significant differences between groups within a species, and error bars show standard deviation.

Minimal inhibitory concentration (MIC) was determined using serial dilution based on the concentration at which 50% of the bacterial growth was inhibited, which is considered one of the basic parameters for evaluating the effectiveness of tested substances [25]. Both AgNPs and the Ag-Cu complex could inhibit microbial growth at a relatively low concentration (Table 2). Optical density measurements showed that the minimal inhibitory concentration reduced microbial growth by approximately half (Figure 2). In the case of CuNPs, a concentration four times higher (25 ppm) was needed to limit the growth of most species. *Salmonella* spp. and *E. coli* were more sensitive, and the inhibitory concentration of CuNPs for these species was twice that of AgNPs and Ag-Cu complex (12.5 ppm for CuNPs and 6.25 ppm for the others).

### 3.3. Membrane Integrity

**Figure 3:**
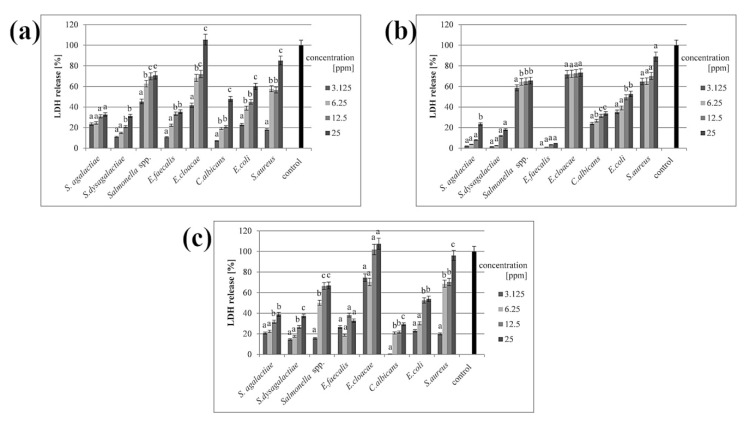
Percentage of LDH release after treatment with (**a**) silver nanoparticles; (**b**) copper nanoparticles; (**c**) silver–copper complex. All samples were measured in triplicate and the results were averaged. Columns labelled a–d indicate statistically significant differences between groups within a species, and error bars show standard deviation.

In all samples where the cell membrane was disturbed, the LDH release was observed in the culture medium. All three types of nanoparticles disrupted the cell membrane, but the greatest effect was observed in the presence of the nanoparticle complex. Results from samples with AgNPs and the complex were slightly similar, while CuNPs caused a weaker disruption of cell membranes. However, all nanomaterials caused disturbance in a dose-dependent manner (Figure 3).

### 3.4. Biofilm Formation

**Figure 4:**
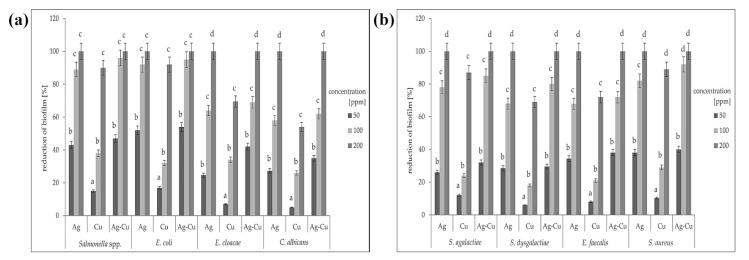
Percentage reduction of the biofilm for each of the test microorganisms treated with different concentrations (ppm) of nanoparticles: (**a**) Gram-negative bacteria and yeast; (**b**) Gram-positive bacteria. All samples were measured in triplicate and the results were averaged. Columns labelled a–d indicate statistically significant differences between all groups within a species, and error bars show standard deviation.

The application of crystal violet dye allowed us to determine the number of microorganisms able to attach to the biotic or abiotic surfaces. The greatest biofilm reduction occurred after treatment with AgNPs and the Ag-Cu complex at a concentration of 200 ppm (Figure 4). This effect was reproducible for all species, including both Gram-positive and Gram-negative bacteria and the yeast *Candida albicans*. No inhibition of biofilm formation was observed when using CuNPs at concentrations of 50 or 100 ppm. Copper nanoparticles at a concentration of 200 ppm did not eliminate the biofilm entirely in any species tested, while silver nanoparticles and the Ag-Cu complex both did. Interestingly, out of the three nanoparticle types, the Ag-Cu complex was most effective at inhibiting biofilm formation, even at the lowest concentration. The results were variable depending on the microorganism species, but in all cases, biofilm reduction was improved with a higher dose concentration.

## 4. Discussion

### 4.1. Antibacterial Properties

We examined the influence of widely used metal nanoparticles on biofilm formation by mastitis pathogens. The analysis shows the high potential of nanomaterials, used both separately and in combination, to treat bovine mastitis, which is currently challenging. Overall, the Ag-Cu complex was more effective than either individual nanoparticle at inhibiting biofilm formation by mastitis pathogens.

Both AgNPs and CuNPs have great potential as antibacterials for inhibiting microbial growth [26]. The combination of AgNPs and CuNPs results in a synergy of effects, even though the activity of separate nanoparticles (Ag or Cu) is distinct. This means that the Ag-Cu complex is more promising than either nanoparticle used alone [27]. A similar effect has been observed in an earlier study on mastitis pathogens, where AgNPs and CuNPs caused a high degree of disruption to microbial viability [28]. We also found that the Ag-Cu complex caused the greatest biofilm reduction (Figure 4). In all samples, even the lowest concentration of the complex (50 ppm) disrupted biofilm growth more than either individual component. In all samples, higher nanomaterial concentration caused greater biofilm reduction. Interestingly, this was true not only for every nanomaterial but also for every microorganism species (Figure 4).

The antibacterial properties of AgNPs and CuNPs are associated with various factors, including their stability in hydrocolloids. Stable nanoparticle hydrocolloids tend not to agglomerate; their surface area is not limited, resulting in stronger antibacterial properties [29]. This was observed in our studies, where AgNPs and the Ag-Cu complex were more stable than CuNPs, and also had better antibacterial properties (Table 1). CuNPs had a zeta potential value of −0.463 mV, whereas AgNPs reached −26.7 mV, which is close to the limit value of colloidal stability (±30 mV). The value of the Ag-Cu complex was closer to the limit than that of CuNPs (−9.09 mV vs. −0.463 mV).

In toxicological studies, certain molecular characteristics must be considered (e.g., shape or size), since they determine the impact of a nanomaterial on the in vitro model [30]. It is assumed that small nanoparticles penetrate deeper into cell structures, but agglomerates, which reach a much greater average size, may have weaker interactions with cells [31]. Therefore, the low toxicity of CuNPs might result from their tendency to form large agglomerates, while the other two nanomaterials did not agglomerate and had a smaller diameter (Figure 1). Although the CuNPs hydrocolloid was composed of two fractions, the average diameter was 345.6 nm, indicating that there were more agglomerates than small nanoparticles (smaller than 100 nm). The toxicity of the nanoparticles is probably determined by their size, not their shape, since all the nanoparticles used had a similar shape (Figure 1). TEM analysis of nanoparticle shape yielded similar results to those reported by Paszkiewicz et al. in 2016 [32], where the shape was also found to be spherical.

According to one of the latest reports, a combination of copper and silver nanoparticles can be used in antibacterial therapies, although the main mode of action is related not to bacterial species, but to cell type and growth, or to nanoparticle uptake ability [33]. Our results confirm that cellular response does not depend on species, which is illustrated in the MIC analysis, where there were no clear distinctions in the reduction of viability of Gram-positive bacteria, Gram-negative bacteria, and yeasts (Table 2, Figure 2). However, in terms of the reduction of biofilm formation, two Gram-negative species (*Salmonella* spp. and *E. coli*) seemed to the most sensitive to the nanoparticles. For other species, biofilm reduction was lower, but for the yeast *C. albicans*, the reduction was similar to that for *E. cloacae* (Figure 4). This is probably the result of the high capacity of both species for developing bacterial resistance [34,35]. The greater sensitivity of Gram-negative bacteria can be explained by their cell wall structure. Their cell walls are much thinner than those of Gram-positive bacteria, despite the presence of an external layer [36].

### 4.2. Possible Mechanisms

The antibacterial properties of nanomaterials result mainly from the generation of free radicals, which disturb the cell wall, membrane, or organelles of bacterial cells [37]. However, there are other mechanisms of microbial cell inactivation. Some of the commonly reported ones include the disruption of intracellular ATP, damage to DNA structures, and damage to other organelles [29]. It is known that metal nanoparticles kill major Gram-positive and Gram-negative pathogens, and that they penetrate and eradicate biofilms; however, the precise mechanism is not fully understood [2]. The interaction of metal nanoparticles with bacterial cells is very complicated due to the enormous number of characteristics that nanoparticles exhibit and to the fact that their mechanism of interaction is still poorly understood. There are many plausible hypotheses for the interactions between nanoparticles and biofilms. These interactions take place on several levels, including disturbing the cell layer and producing reactive oxygen species that damage internal structures [19]. Silver ions, which are generated from silver nanoparticles, bind to the negatively-charged layer, causing cell perforation and cell death [38]. The accumulation of metal nanoparticles around bacterial cells and in biofilm networks has been visualised in a previous study [39]; the effect was dependent on nanoparticle type. Larger nanoparticles (more than 10 nm in diameter) interact with the cell wall or membrane [21]. In our research, the nanoparticles and their complex agglomerates had a diameter over 10 nm (AgNPs: 154.1 nm; CuNPs: 345.6 nm; Ag-Cu complex: 174.2 nm); therefore, they attacked the internal parts of cell. Furthermore, even if nanoparticles or their agglomerates are too large to penetrate through the entire biofilm, they interact with planktonic cells, which still reduces biofilm formation. Dispersion is a critical step in biofilm formation because under natural conditions it allows cells to spread to new areas [4]. By attacking planktonic cells, nanoparticles prevent this spread.

In our research, LDH release was dose-dependent in all samples (Figure 3), which supports our hypothesis about the interaction of nanomaterials with the outer part of microbial cells. The mechanism of this interaction is well-known, but biofilms show entirely different phenotypes from planktonic forms [40]. The penetration of antimicrobials and their impact on microbial cells in biofilms is hampered, mainly due to the presence of exopolysaccharide (EPS), which presumably binds directly to antimicrobial agents [40]. The presence of EPS and the complex structure of biofilms contribute to the acquisition of resistance, and thus make treatment of infections more difficult [9]. This is affected by the location of cells in the biofilm, where metabolically active cells are located in the other parts of the structure [5], and this microbial heterogeneity makes it possible for resistance characteristics to spread throughout the entire biofilm [3]. It is believed that nanomaterials may damage signalling molecules, leading to the inhibition of gene expression pathways required to develop and modify the biofilm structure. This can cause the biofilm to lose its resistant traits [41].

The positive effects of metal nanoparticles on the inhibition of biofilms made up of certain bacteria species have been observed in research by Gurunathan et al. [42], who suggest that AgNPs may constitute an adjuvant for curing bacterial infections. The same was demonstrated by Martinez-Gutierrez et al. [43], who found that AgNPs not only inhibited biofilm formation, but also induced cell death.

Thanks to their high antibacterial potential, nanoparticles are considered one of the most promising agents for preventing bovine mastitis [44,45,46]. However, despite their excellent properties, a great number of aspects must be considered before use, such as their influence on mammalian tissues and on whole organisms [47]. Nevertheless, the proposed solution for treating mastitis infections might alleviate serious hazards for animals, entrepreneurs, and the human population [10,11].

## 5. Conclusions

This research (and its possible follow-up studies) proposes a promising treatment for mastitis, an illness caused to a large extent by biofilm formation by microorganisms. The presented results show that metal nanoparticles are able to disrupt the biofilm. Particularly noteworthy is the combination of AgNPs and CuNPs, which yielded the best results. These results are important, especially since the threat of mastitis may be more serious than it seems at first glance.

## Figures and Tables

**Table 1:** Physicochemical parameters (average hydrodynamic diameter, zeta potential, and structure) of nanoparticles used (Ag, silver nanoparticles; Cu, copper nanoparticles; Ag-Cu, silver–copper complex).

Nanomaterial	Average Hydrodynamic Diameter (nm)	Zeta Potential (mV)	Structure
Ag	154.1	−26.7	spherical
Cu	345.6	−0.463	spherical
Ag-Cu	174.2	−9.09	spherical

**Table 2:** Values of minimal inhibitory concentration (ppm) of the nanoparticles used (Ag, silver nanoparticles; Cu, copper nanoparticles; Ag-Cu, silver–copper complex) for each microorganism strain.

Nanomaterial	*S. agalactiae*	*S. dysagalactiae*	*Salmonella* spp.	*E. faecalis*	*E. cloacae*	*C. albicans*	*E. coli*	*S. aureus*
Ag	3.125	6.25	6.25	6.25	6.25	6.25	6.25	12.5
Cu	25	25	12.5	25	25	25	12.5	25
Ag-Cu	3.125	6.25	6.25	6.25	6.25	6.25	6.25	12.5

## Data Availability

The data presented in this study are available on request from the corresponding author.
